# Prospective Study of ADAMTS13 and von Willebrand Factor’s Role in the Prediction of Outcomes in Acute Ischemic Stroke

**DOI:** 10.3390/jcm14072470

**Published:** 2025-04-04

**Authors:** Michail Makris, Eleni Gavriilaki, Eleftheria Ztriva, Paschalis Evangelidis, Elmina Lefkou, Efthymia Vlachaki, Stavroula Bountola, Vasileios Perifanis, Miltiadis Matsagkas, Christos Savopoulos, Georgia Kaiafa

**Affiliations:** 1 First Propaedeutic Department of Internal Medicine, AHEPA University General Hospital of Thessaloniki, Aristotle University of Thessaloniki, 54636 Thessaloniki, Greece; michaelmakris4@gmail.com (M.M.); elztriva@gmail.com (E.Z.); bountolastavroula@gmail.com (S.B.); perifanisb@auth.gr (V.P.); chrisavopoulos@gmail.com (C.S.); gdkaiafa@yahoo.gr (G.K.); 2 Second Propedeutic Department of Internal Medicine, Aristotle University of Thessaloniki, 54642 Thessaloniki, Greece; pascevan@auth.gr; 3 Hematology-Transfusion Medicine Department, Larissa University Hospital, Faculty of Medicine, University of Thessaly, 41110 Larissa, Greece; elmina.lefkou@thrombosis.gr; 4 Hematological Laboratory, Second Department of Internal Medicine, Hippocration General Hospital, Aristotle University of Thessaloniki, 54642 Thessaloniki, Greece; efivlachaki@auth.gr; 5 Department of Vascular Surgery, Larissa University Hospital, Faculty of Medicine, University of Thessaly, 41110 Larissa, Greece; milmats@gmail.com

**Keywords:** ADAMTS13, disability, modified Rankin scale, NIHSS score, stroke, thrombosis, von Willebrand factor

## Abstract

**Background:** In this prospective study, the prognostic role of ADAMTS13 activity and von Willebrand (VWF) antigen (VWF: Ag) levels in ischemic stroke outcomes was investigated. **Methods:** Patients diagnosed with acute ischemic stroke were prospectively enrolled in this study, while samples for ADAMTS13 activity and VWF: Ag level measurements were collected upon their admission to our unit. The National Institutes of Health Stroke Scale (NIHSS) score was estimated upon admission and at discharge. The modified Rankin scale for neurologic disability (Rankin) score was estimated based on the patient’s history before the stroke onset, during admission (RankinAdm), and at discharge (RankinDis). **Results:** In the study, 29 patients with a median age of 82.5 (51, 92) were included. In univariate analysis, ADAMTS13 activity during admission was associated with platelet values at the same time point (r = 0.12, *p* = 0.01) and VWF: Ag levels were associated with age (r = 0.439, *p* = 0.04), previous ischemic stroke (r = 0.9176, *p* = 0.031), and glucose levels (r = 0.64, *p* = 0.049). Associations between ADAMTS13/VWF: Ag Ratio with RankinDis (r = 0.3253, *p* = 0.03), and the change between RankinDis and RankinAdm (r = 0.1589, *p* = 0.014) were identified. Additionally, VWF: Ag levels during admission were correlated with RankinDis (r = 0.0072, *p* = 0.049). **Conclusions:** These markers might be useful as biomarkers for the prediction of poor outcomes after stroke.

## 1. Introduction

Stroke is considered the second cause of death worldwide, with a record of 5.5 million deaths annually, while 50% of the surviving patients suffer from severe disabilities [1]. Acute ischemic stroke accounts for about 85–90% of stroke cases worldwide. Intravenous (IV) thrombolysis with IV tissue plasminogen activator (t-PA) within the first 4.5 h after ischemic stroke and reperfusion therapies, such as mechanical thrombectomy, have resulted in a reduction in mortality and long-term disability rates in these patients [2]. Despite the therapeutic advances made, there are still limitations in other patients. Thus, further research is of paramount importance to understand the pathophysiological mechanisms and causes of stroke, as well as the development of new predictive tools and therapeutic strategies [3].

Von Willebrand factor (VWF) is a multimeric plasma glycoprotein that plays an important role in primary hemostasis as it promotes platelet adhesion, leading to clot formation and stabilization of factor VII (FVII) [4]. Simultaneously, ADAMTS13 (a disintegrin and metalloproteinase with a thrombospondin type 1 motif, member 13), a metalloprotease from the ADAMTS family, is responsible for the regulation of VWF as it cleaves the ultra-large polymers of VWF into smaller and less active molecules [5]. ADAMTS13 interacts with both soluble VWF and endothelium-bound ultra-large VWF (ULVWF) strings, leading to the cleavage of these ULVWF structures [6]. This cleavage is enhanced by fluid shear stress, which suggests that the ULVWF on cell surfaces is in an “open” conformation, making it more vulnerable to ADAMTS13 cleavage. In contrast, when ULVWF is released into the bloodstream as a soluble form, it adopts a “closed” conformation, which is resistant to ADAMTS13 cleavage unless arterial shear stress is developed. Moreover, the binding of platelet glycoprotein 1b or coagulation factor VIII (FVIII) to soluble VWF significantly accelerates its cleavage by ADAMTS13 under shear conditions. These interactions play a crucial role in thrombotic processes, especially in the cerebral vasculature, as dysregulation of this pathway has been implicated in thrombotic microangiopathies (TMAs) and, increasingly, in ischemic stroke [7,8]. Decreased ADAMTS13 is a diagnostic hallmark in thrombotic thrombocytopenic purpura, a TMA, characterized by hemolytic anemia, thrombocytopenia, and thromboses in small vessels, and high mortality and morbidity [9]. Moreover, decreased activity of ADAMTS13 has been recognized in several other clinical conditions, such as HELLP syndrome (hemolysis, elevated liver enzymes, and low platelets) and drug-induced TMAs [10,11].

Key research findings indicate that the VWF/ADAMTS13 axis might have a role in the acute ischemic stroke development and outcomes prediction in these patients [12]. It has been suggested that in the general population, ADAMTS activity, VWF: Ag, and especially the VWF: Ag/ADAMTS13 Ratio, are associated with stroke risk development independently of other factors [13]. However, data regarding the role of these markers in the ischemic stroke prognosis of real-world patients are conflicting [13]. In the era of precision medicine, accurate prognosis of ischemic stroke outcomes—given the increased disease burden—is of paramount importance. Thus, the aim of this study is to investigate the correlation between ADAMTS13 activity and VWF antigen (VWF: Ag) levels in the outcomes, severity, and disability degree in patients with ischemic stroke.

## 2. Materials and Methods

### 2.1. Study Design and Population

In this prospective study, consecutive adult patients (age ≥ 18 years) diagnosed with acute ischemic stroke at the 1st propedeutic Department of Internal Medicine of AHEPA hospital, between January 2020–July 2024, were enrolled. All study participants provided informed consent, and the study was performed according to the Declaration of Helsinki principles. The study was approved by the institutional board of AHEPA Hospital (Greece 580/25.6.2020). Inclusion criteria were the following:

- Age ≥ 18 years
- Onset of acute ischemic stroke within 24 h of their presentation to our department. The acute onset of focal neurologic deficits with radiological evidence confirmation was essential for the diagnosis of ischemic stroke or transient ischemic attack (TIA). In all the study participants, computed tomography or magnetic resonance imaging scans were carried out within 4 weeks of the onset of neurological deficits. TIAs were diagnosed based on the duration of symptoms up to 24 h from the onset and the absence of confirmed damage on neuroimaging.

Patients with known hematological disorders or other TMAswere not included. Upon admission to our unit, all patients underwent full clinical (with comprehensive clinical examination and medical history recording), laboratory (complete blood count, coagulation, and biochemistry tests), and radiological evaluation. Data regarding their sex, age, and medical history of hypertension, diabetes mellitus, dyslipidemia, atrial fibrillation, peripheral vascular disease, coronary artery disease, and current smoking status were obtained. Additionally, laboratory values upon admission (platelet [PLT], low-density lipoprotein [LDL], high-density lipoprotein [HDL], hemoglobin A1C [HbA1c], and glucose), as well as systolic (SBP) and diastolic (DBP) blood pressure and heart rate (HR), were recorded. BP was calculated as the mean of the last two measurements on a total of three measurements using a certified upper arm blood pressure monitor device [14].

The National Institutes of Health Stroke Scale (NIHSS) score was estimated both during admission (NIHSSadm) and at discharge (NIHSSdis), while the change between NIHSSadm and NIHSSdis was calculated for each patient [15]. The modified Rankin scale for neurologic disability (Rankin) score was estimated based on the patient’s history before the stroke onset (RankinPre), during admission (RankinAdm), and at discharge (RankinDis) [16]. The change between RankinAdm and RankinPre scores, and RankinDis and RankinAdm were calculated. Furthermore, upon admission to our unit, patients’ samples for the measurement of ADAMTS13 and VWF: Ag were collected, before the application of any therapeutic measure, aliquoted rapidly, and stored at −80 °C immediately.

### 2.2. Biochemical Assays

For the measurement of ADAMTS13 plasma activity, a commercially available enzyme-linked immunosorbent sandwich assay (ELISA) test kit (Technozym^®^, catalog number: 5450701), as previously described, was used [17]. VWF antigen (VWF: Ag) levels were measured with an immunoturbidimetric assay with the Siemens Healthineers BCS^®^ XP System (catalog number: 10445967). The ratio of VWF: Ag to ADAMTS13 activity was calculated.

### 2.3. Statistical Analysis

Statistical analysis of the data was performed with SPSS 28.0 (IBM SPSS Statistics for Windows, Version 28.0. Armonk, NY, USA: IBM Corp). For the presentation of variables with a normal distribution, means with standard deviations (SDs) were used, while for those with non-normal distribution, medians with minimum (min) and maximum (max) values were used. Assessment of normality was carried out with the Shapiro–Wilk test. Moreover, categorical variables were presented as frequencies and percentages. Nonparametric related variables were compared with the Wilcoxon signed-rank test. Moreover, the Mann–Whitney test for comparing unrelated nonparametric variables was performed between the groups. A logarithmic transformation of a non-to-normal distribution was performed when considered essential. Pearson’s and Spearman’s rank correlation coefficients, for parametric and nonparametric variables, respectively, were employed for the univariate analysis.

## 3. Results

### 3.1. Clinical and Laboratory Characteristics of Study Participants

In this study, 29 patients (17 females and 12 males) were included, with a median age of 82.5 (51, 92) years, diagnosed with ischemic stroke, while 11 (37.9%) of them suffered from TIA. Moreover, 19 patients (65.5%) had a previous history of arterial hypertension, 8 (27.6%) had diabetes mellitus, 8 (27.6%) had dyslipidemia, 11 (37.9%) had atrial fibrillation, 2 (6.9%) had peripheral vascular disease, and 8 (27.6%) had coronary artery disease. Additionally, nine (31%) reported a history of a previous ischemic stroke. Eight (27.6%) study participants were smokers, and one (3.4%) had a history of alcohol abuse. The clinical and laboratory characteristics of the study population are presented in Table 1. Moreover, none of our patients were treated with IV thrombolysis or thrombectomy.

### 3.2. Ischemic Stroke Severity

The median hospitalization days were 7 (2, 20), while the NIHSSadm and NIHSSdis scores were 5 (1, 23) and 3 (0, 20), respectively. The median change between NIHSSadm and NIHSSdis was −1 (−7, 4), while NIHSSdis was significantly lower compared to NIHSSadm (*p* = 0.007). Median RankinPre, RankinAdm, and RankinDis scores were 2 (0, 4), 4 (0, 5), and 3.5 (0, 6), respectively. Additionally, the median change between RankinAdm and RankinPre was 2 (−1, 5), while between RankinDis and RankinAdm it was 0 (−2, 2). RankinAdm was significantly lower compared to RankinPre (*p* < 0.001). A statistically significant difference was not observed between RankinDis and RankinAdm (*p* = 0.166). These findings are summarized in Table 2.

### 3.3. ADAMTS13 Activity, VWF: Ag, and the VWF: Ag/ADAMTS13 Ratio

The median value of ADAMTS13 activity during admission was 77.5% (20.4, 100), while the median levels of VWF: Ag were 232.1% (125.9, 470.0). In univariate analysis, ADAMTS13 activity was not associated with VWF: Ag levels (r = −0.336, 95% CI: −3.12, 0.67, *p* = 0.20). Moreover, the median VWF: Ag/ADAMTS13 Ratio was 3.1 (1.3, 11.4). Additionally, ADAMTS 13 activity [65.6% (48.2–100) versus 77.5% (20.4–100), *p* = 0.877) and VWF: Ag levels [245.8% (125.9–470) versus 227.3% (131.6–430), *p* = 0.604] were not statistically different between patients with TIA and the rest of study participants. At the same time, the median activity of ADAMTS13 in male patients was 63.7% (39–87) versus 78.3% (20.4–100) in female patients (*p* = 0.080). Similarly, a statistical difference was not observed in VWF: Ag levels between male and female study participants [247.5% (129–470) versus 225. 1% (125.9–307), *p* = 0.368].

### 3.4. ADAMTS13 Activity, VWF: Ag, VWF: Ag/ADAMTS13 Ratio, and Associations with Baseline Clinical and Laboratory Characteristics

In univariate analysis, ADAMTS13 activity during admission was associated with PLT values at the same time point (r = 0.12, 95% CI: 0.03, 0.22, *p* = 0.01). Furthermore, VWF: Ag levels were correlated with age (r = 0.439, 95% CI: 0.22, 8.57, *p* = 0.04), previous ischemic stroke (r = 0.9176, 95% CI: 9.19, 174.33, *p* = 0.031), and glucose laboratory values at the same time point (r = 0.64, 95% CI: 0.01, 1.29, *p* = 0.049). VWF: Ag/ADAMTS13 Ratio was not associated with any of the studied factors.

### 3.5. ADAMTS13 Activity, VWF: Ag, VWF: Ag/ADAMTS13 Ratio, and Associations with Stroke Severity Factors

The findings of univariate analysis between ADAMTS13 activity, VWF: Ag, and the ADAMTS13/VWF: Ag Ratio with stroke severity factors are presented in Table 3. The ADAMTS13/VWF: Ag Ratio was associated with RankinDis (r = 0.3253, 95% CI: 0.0339, 0.6167, *p* = 0.03), and the change between RankinDis and RankinAdm (r = 0.1589, 95% CI: 0.0357, 0.2820, *p* = 0.014). Moreover, VWF: Ag levels during admission were correlated with RankinDis (r = 0.0072, 95% CI: 0.0001, 0.0144, *p* = 0.049).

## 4. Discussion

In our real-world prospective study, ADAMTS13 activity, VWF: Ag, VWF: Ag/ADAMTS13 Ratio, and their correlations with ischemic stroke severity indices were examined. We failed to identify a statistically significant association between ADAMTS13 activity and VWF: Ag levels, possibly due to the relatively low number of study participants. Moreover, a correlation of VWF: Ag with the degree of disability at the patient’s discharge (RankinDis) was identified. Similarly, the VWF: Ag/ADAMTS13 Ratio was associated with RankinDis and the change between RankinDis and RankinAdm.

The crucial role of ADAMTS13 and VWF has been identified in the ischemic stroke development. Various experimental and translational studies have examined the potential role of these molecules in the pathogenesis of ischemic stroke. An imbalance between ADAMTS13 and VWF has been proposed as pathogenic in thrombus development [18]. Complete genetic deficiency of ADAMTS13 in mice has been found to induce a prothrombotic state [19]. Moreover, ADAMTS13 has an important role in stroke angiogenesis, by induction of vascular endothelial growth factor production [20]. Stoll et al. recognized rare variants in the ADAMTS13 gene in pediatric patients in their study, which were associated with the development of ischemic stroke [21]. In the experimental study of Zhao and colleagues, it was shown that infusion of recombinant human ADAMTS13 before reperfusion in mice with ischemic stroke resulted in a significantly reduced infarct volume and was associated with improved functional outcomes [22]. Fujioka et al. reported that in mice with ADAMTS13 gene depletion (−/−) and ischemic stroke, after reperfusion, the blood flow in the ischemic region was significantly lower in comparison to the wild-type mice (*p* < 0.05). Concurrently, in these mice, the volume of the infarct after 24 h was significantly greater (*p* < 0.01) [23]. The absence of ADAMTS13 in mice with ischemic stroke has been correlated with increased myeloperoxidase activity and expression of pro-inflammatory cytokines such as interleukin-6 and tumor necrosis factor-α [24]. Thus, reduced ADAMTS13 activity, as shown by both functional and genetic data, might result in prothrombotic and pro-inflammatory conditions, which contribute to brain ischemia.

Based on the above hypothesis, several research groups have compared ADAMTS13 activity and VWF levels between healthy controls and ischemic stroke patients. Bongers and colleagues in their case–control study of 124 patients with ischemic stroke and 125 matched controls, found that VWF: Ag levels were significantly higher in stroke patients in comparison to controls (*p* = 0.002) [18]. In our study, however, a control group was not included; thus, it was not possible to perform such a direct comparison. In the study by Hanson et al., the levels of VWF over a 3-month period after the ischemic stroke were significantly increased in large-vessel disease, cardioembolic, and cryptogenic stroke, in comparison to healthy controls, but not in patients with small-vessel disease stroke [25]. Interestingly, in the Rotterdam study, a population-based cohort study with 6130 participants aged >55 years, both ADAMTS13 activity and VWF: Ag levels were correlated with a great risk of cardiovascular mortality [26]. Future studies, with long-term follow-up, are essential to evaluate the prognostic role of these markers in the prediction of cardiovascular mortality in acute ischemic stroke survivors. The CHA2DS2-VASc Score (congestive heart failure (1 point), hypertension, age ≥ 75 years [2 points], diabetes mellitus, previous stroke/transient ischemic attack [2 points], vascular disease, age 65–74 years, and female gender) has been established for the estimation of stroke prediction in patients with atrial fibrillation [27]. Zhang et al. have identified correlations between both ADAMTS13 activity and VWF and the CHA2DS2-VASc Score in patients with and without atrial fibrillation [28]. Grosse et al. reported that ADAMTS-13 activity was lower in patients with ischemic stroke related to atrial fibrillation compared to patients with stroke of undetermined cause (*p* = 0.0227) [29].

Impaired function of ADAMTS13 might contribute to the development of cardiovascular disease. In the case–control study by Bongers and colleagues, it was shown that patients with cardiovascular disease had lower levels of ADAMTS13 activity in comparison to healthy individuals [30]. Furthermore, in the observational study of Denorme et al., it was reported that those with acute ischemic stroke had reduced ADAMTS13 activity compared to healthy volunteers [31]. Similar results were also obtained for patients with chronic cerebrovascular disease [31]. Additionally, in this study, the VWF: ADAMTS13 ratio was associated with stroke severity and mortality [31]. In our analysis, VWF: Ag levels were correlated with disability degree estimated with a modified Rankin scale at discharge. Similarly, genetic and functional data have been reported in pediatric patients regarding decreased ADAMTS13 and stroke development [32,33]. Su et al. in their prospective study, investigated the potential associations between baseline ADAMTS13 antigen values and outcomes 90 days post-thrombolysis in 163 patients (median age: 66.2) with ischemic stroke [34]. However, they failed to identify ADAMTS13 antigen levels, assessed before thrombolysis, as independent predictors for 90-day clinical outcomes. Future research should focus on the role of ADAMTS13 genetic variants and stroke severity and mortality.

Qu et al. have shown that in patients with cerebral infarction, the ADAMTS13 administration resulted in reduced expression of inflammatory molecules such as intercellular cell adhesion molecule-1 and matrix metalloproteinase-9, suggesting the endothelial- and neuroprotective role of ADAMTS13 [35]. Nakano et al., in their experimental study in mice with ischemic stroke, have shown that the therapeutic window of recombinant ADAMTS13 administration is higher compared to tissue-type plasminogen t-PA thrombolysis [36]. Denorme et al., in their experimental study, also reported the thrombolytic activity of recombinant ADAMTS13 in ischemic stroke, which also enhanced the thrombolytic activity of t-PA [37]. McCabe et al. have shown in their prospective case–control study that ADAMTS13 activity is reduced in the early period (≤4 weeks) after the stroke in patients who receive aspirin [38]. More data regarding the role of recombinant ADAMTS13 in the management of ischemic stroke are essential to better understand the safety and efficacy of this approach in clinical practice. Multicenter collaboration and a prospective study design are crucial for this aim.

Prochazka et al. have reported that increased levels of ADAMTS13, as assessed before the endovascular procedure, were associated with poor clinical outcomes and modified Rankin scale scores 3 to 6 [7]. Similarly, in the study by Taylor and colleagues, the VWF: Ag/ADAMTS13 activity ratio at stroke presentation correlated with higher modified Rankin scale scores (*p* = 0.021) and NIHSS scores (*p* = 0.029) at follow-up [39]. We have shown that both VWF: Ag and the VWF: Ag/ADAMTS13 activity ratio correlated with modified Rankin scale scores, but we failed to identify associations between ADAMTS13 activity and patients’ outcomes. This can be attributed to the relatively low number of study participants.

High circulating VWF: Ag concentrations have been linked to the no-reflow phenomenon and increased microvascular resistance, which could hinder successful reperfusion despite large-vessel recanalization, not only in patients with ischemic stroke but also with acute myocardial infarction [40]. It would be interesting to examine the possible associations between this marker and microvascular dysfunction in these patients. For this aim, a non-interventional study of microcirculation dynamics in acute ischemic stroke survivors during long-term follow-up can be helpful [41]. In some prior published studies, it was suggested that reduced ADAMTS13 activity is associated with larger infarct sizes and lower recanalization rates after thrombectomy [42]. Future studies in this field should focus on the investigation of possible correlations between ADAMTS13 activity and VWF: Ag levels with infarct volume, as measured by ASPECTS (Alberta stroke program early CT score) scoring or DWI (diffusion-weighted imaging) lesion volumetry in acute ischemic stroke patients.

Several limitations should be recognized in our study. Firstly, it was a monocenter study, limiting the generalizability of our results. Moreover, the patient’s sample was relatively small, while a control group was not included; thus, it was not possible to compare ADAMTS13 activity and VWF: Ag levels between our patients and healthy controls. Additionally, due to the sample size, we did not compare the levels of these markers between the different stroke etiologies; furthermore, data regarding infarct volume were not available. Another limitation of our study is that our study population mainly consisted of elderly adults, and given that aging is associated with an increase in clotting factors such as fibrinogen, factor VII, factor VIII, and VWD, resulting in a higher tendency for blood clot formation, the generalizability of our data might be restricted. More studies in this field, including also younger individuals with acute ischemic stroke, are of paramount importance. Finally, in our study, long-term follow-up of our patient population was not performed. In the future, we plan to reach our study participants, assess their status (alive or dead, and cause of death), and degree of disability using the modified Rankin score. Thus, the associations between ADAMTS13 activity and VWF: Ag levels at baseline and long-term outcomes will be evaluated.

## 5. Conclusions

In this prospective study, the association between ADAMTS13 activity, VWF: Ag, and the VWF: Ag/ADAMTS13 Ratio and stroke outcomes was examined. Based on our results, a direct relationship between ADAMTS13 and the severity of ischemic stroke was not found. However, a correlation of VWF: Ag levels and the VWF: Ag/ADAMTS13 Ratio with the disability rate at the patient’s discharge, as evaluated with a modified Rankin scale, was identified. Thus, these markers might be useful as biomarkers for the prediction of poor outcomes after a stroke. Larger and multicenter studies are essential to establish the prognostic role of these markers in everyday clinical practice.

## Figures and Tables

**Table 1:** Clinical and laboratory characteristics of study participants.

N = 29	
Median age (min, max)	82.5 (51, 92)
Sex	
Male, N (%)	12 (41.4%)
Female, N (%)	17 (58.6%)
TIA, N (%)	11 (37.9%)
Hypertension, N (%)	19 (65.5%)
Diabetes mellitus, N (%)	8 (27.6%)
Dyslipidemia, N (%)	8 (27.6%)
Atrial fibrillation, N (%)	11 (37.9%)
PVD, N (%)	2 (6.9%)
CAD, N (%)	8 (27.6%)
Current smoker, N (%)	8 (27.6%)
Alcohol abuse, N (%)	1 (3.4%)
Mean PLT counts (×10^9^/L) ± SD	215.7 ± 76.5
Mean LDL (mg/dL) ± SD	90.9 ± 76.5
Median HDL (mg/dL) (min, max)	41.3 (20, 91)
Median HbA1c (%) (min, max)	5.7 (4.8, 9.1)
Mean Glucose mg/dL ± SD	136.6 ± 62.7
Mean SBP (mmHg) ± SD	148.8 ± 32.8
Median DBP (mmHg) (min, max)	75 (55, 132)
Mean HR ± SD	79 ± 14.2

TIA: transient ischemic attack, PVD: peripheral vascular disease, CAD: coronary artery disease, PLT: platelet, L: liter, SD: standard deviation, LDL: low density lipoprotein, mg: milligram, dL: deciliter, HDL: high density lipoprotein, HbA1c: hemoglobin A1C, SBP: systolic blood pressure, DBP: diastolic blood pressure, mmHg: millimeter of mercury, HR: heart rate.

**Table 2:** Stroke severity parameters.

Variable	Median (min, max)
Hospitalization days	7 (2, 20)
NIHSSadm	5 (1, 23)
NIHSSdis	3 (0, 20)
Change between NIHSSadm and NIHSSdis	−1 (−7, 4)
RankinPre	2 (0, 4)
RankinAdm	4 (0, 5)
RankinDis	3.5 (0, 6)
Change between RankinAdm and RankinPre	2 (−1, 5)
Change between RankinDis and RankinAdm	0 (−2, 2)

NIHSS: National Institutes of Health Stroke Scale/Score; NIHSSadm: NIHSS estimated at admission; NIHSSdis: NIHSS estimated at discharge; Rankin: Rankin score; RankinPre: Rankin score estimated before stroke onset; RankinAdm: Rankin score estimated during admission; RankinDis: Rankin score estimated during discharge.

**Table 3:** Univariate analysis between ADAMTS13 activity, VWF: Ag, ADAMTS13/VWF: AgVWF: Ag Ratio, and stroke severity factors.

	ADAMTS13 Activity			VWF: Ag				VWF: Ag/ADAMTS13 Ratio	
	r	95% CI	*p*	r	95% CI	*p*	r	95% CI	*p*
Hospitalization days	0.0168	−0.0808, 0.1144	0.72	0.0025	−0.0171, 0.0222	0.792	0.1449	−0.6587, 0.9485	0.713
NIHSSadm	−0.0028	−0.1315, 0.1259	0.96	−0.0046	−0.0333, 0.0241	0.744	0.0081	−1.1714, 1.1875	0.983
NIHSSdis	−0.0040	−0.1367, 0.1287	0.95	0.0009	−0.0328, 0.0346	0.955	0.1690	−1.0972, 1.4353	0.784
Change between NIHSSadm and NIHSSdis	0.0022	−0.0417, 0.0461	0.91	0.0012	−0.0099, 0.0122	0.828	0.0379	−0.3785, 0.4543	0.852
RankinPre	−0.0059	−0.0321, 0.0203	0.64	0.0034	−0.0021, 0.0089	0.211	0.1228	−0.1044, 0.36501	0.276
RankinAdm	−0.0096	−0.0410, 0.0217	0.53	0.0057	−0.0004, 0.0119	0.065	0.1664	−0.0944, 0.4272	0.2
RankinDis	−0.0194	−0.0550, 0.0161	0.27	0.0072	0.0001, 0.0144	0.049	0.3253	0.0339, 0.6167	0.03
Change between RankinAdm and RankinPre	−0.0037	−0.0370, 0.0295	0.81	0.0023	−0.0041, 0.0087	0.461	0.0436	−0.2203, 0.3704	0.736
Change between RankinDis and RankinAdm	−0.0098	−0.0250, 0.0054	0.19	0.0014	−0.0019, 0.0048	0.389	0.1589	0.0357, 0.2820	0.014

ADAMTS13: a disintegrin and metalloproteinase with a thrombospondin type 1 motif, member 13; VWF: Ag: von Willebrand factor antigen; NIHSS: National Institutes of Health Stroke Scale/Score; NIHSSadm: NIHSS estimated during at admission; NIHSSdis: NIHSS estimated during at discharge; Rankin: Rankin score; RankinPre: Rankin score estimated during before stroke onset; RankinAdm: Rankin score estimated at admission; RankinDis: Rankin score estimated at discharge.

## Data Availability

The data presented in this study are not available due to ethical reasons.
